# Licochalcone B Induces ROS-Dependent Apoptosis in Oxaliplatin-Resistant Colorectal Cancer Cells via p38/JNK MAPK Signaling

**DOI:** 10.3390/antiox12030656

**Published:** 2023-03-07

**Authors:** Ah-Won Kwak, Woo-Keun Kim, Seung-On Lee, Goo Yoon, Seung-Sik Cho, Ki-Taek Kim, Mee-Hyun Lee, Yung Hyun Choi, Jin-Young Lee, Jin Woo Park, Jung-Hyun Shim

**Affiliations:** 1 Biosystem Research Group, Department of Predictive Toxicology, Korea Institute of Toxicology, Daejeon 34114, Republic of Korea; 2 Department of Biomedicine, Health & Life Convergence Sciences, BK21 Four, College of Pharmacy, Mokpo National University, Muan 58554, Republic of Korea; 3 Department of Pharmacy, College of Pharmacy, Mokpo National University, Muan 58554, Republic of Korea; 4 College of Korean Medicine, Dongshin University, Naju 58245, Republic of Korea; 5 Department of Biochemistry, College of Korean Medicine, Dong-Eui University, Busan 47227, Republic of Korea; 6 Department of Biological Sciences, Keimyung University, Daegu 42601, Republic of Korea; 7 The China-US (Henan) Hormel Cancer Institute, Zhengzhou 450008, China

**Keywords:** apoptosis, colorectal cancer, JNK/p38 MAPK, Licochalcone B, reactive oxygen species

## Abstract

Licochalcone B (LCB) exhibits anticancer activity in oral cancer, lung cancer, and hepatocellular carcinoma cells. However, little is known about its antitumor mechanisms in human oxaliplatin-sensitive and -resistant colorectal cancer (CRC) cells. The purpose of the present study was to investigate the antitumor potential of LCB against human colorectal cancer in vitro and analyze its molecular mechanism of action. The viability of CRC cell lines was evaluated using the MTT assay. Flow cytometric analyses were performed to investigate the effects of LCB on apoptosis, cell cycle distribution, reactive oxygen species (ROS), mitochondrial membrane potential (MMP) dysfunction, and multi-caspase activity in CRC cells. The results demonstrated that LCB induced a reduction in cell viability, apoptosis, G2/M cell cycle arrest, ROS generation, MMP depolarization, activation of multi-caspase, and JNK/p38 MAPK. However, p38 (SB203580) and JNK (SP600125) inhibitors prevented the LCB-induced reduction in cell viability. The ROS scavenger N-acetylcysteine (NAC) inhibited LCB-induced reduction in cell viability, apoptosis, cell cycle arrest, ROS generation, MMP depolarization, and multi-caspase and JNK/p38 MAPK activities. Taken together, LCB has a potential therapeutic effect against CRC cells through the ROS-mediated JNK/p38 MAPK signaling pathway. Therefore, we expect LCB to have promising potential as an anticancer therapeutic and prophylactic agent.

## 1. Introduction

Chemotherapy is one of the most effective treatments for patients with colorectal cancer (CRC). FOLFOX therapy (composed of fluorouracil, folinic acid, and oxaliplatin (Ox)) is a classic first-line treatment for CRC [1]. Ox-based chemotherapy is a widely used treatment strategy in patients with stage 2 and 3 CRC after surgical resection [2]; however, Ox resistance is a major cause of treatment failure, resulting in cancer recurrence and metastasis to other organs [3]. Ox interacts with nucleotides in DNA strands to form crosslinks that prevent DNA synthesis and replication, inducing apoptosis in cancer cells [4]. Associated with these interactions, DNA repair protein complexes that restore mismatches or abnormal nucleotides in DNA strands frequently have an effect on the efficacy and safety of Ox [4]. Therefore, there is an urgent need to identify the underlying mechanisms and ways to reverse/sensitize Ox resistance in CRC treatment.

Natural products are being underscored as a source of various pharmacological constituents for the treatment of various disorders such as infectious diseases, malignant tumors, and neurological conditions [1,5]. Ongoing studies on natural products have led to approximately 50% of the currently available cancer treatments being derived directly or indirectly from natural constituents [6]. Therefore, natural products may be a potential drug source for the treatment of CRC.

Chalcone, one of the major secondary metabolites of plants belonging to the flavonoid family, can act on various drug targets through exhibiting structural heterogeneity [7,8]. Compounds with chalcone-based structures and/or templates exert pharmacological activities, such as anti-cancer, anti-inflammatory, anti-diabetic, anti-oxidant, and anti-microbial effects, and exhibit diverse biological potential [7,8,9]. Licochalcone B (LCB), a member of the chalcone family, is a natural compound derived from the roots of the Chinese medicinal herb *Glycyrrhiza inflata* [8]. According to previous reports, LCB showed anticancer efficacy in skin cancer, hepatoma, oral cancer, bladder cancer, and lung cancer through cell cycle arrest, intrinsic and extrinsic apoptosis pathways, and apoptosis pathways, with EGFR and MET as dual targets [10,11,12,13,14]. However, the exact molecular mechanisms underlying the anticancer effects of LCB in CRC cells have not yet been elucidated.

Although carcinogenesis and apoptosis are conflicting phenomena, reactive oxygen species (ROS) have been reported to play a significant role in both [15]. ROS, either oxygen-derived free radicals (e.g., superoxide anions and hydroxyl radicals) or non-radical molecules (e.g., hydrogen peroxide), are short-lived and highly reactive small molecules [16]. Low doses of ROS are associated with the induction of cell survival mechanisms, such as proliferation, differentiation, and cell cycle progression, whereas high doses activate apoptotic molecular processes mediated by mitochondria, death receptors, the endoplasmic reticulum (ER), and mitogen-activated protein kinases (MAPKs) [15,16]. Several studies have shown that apoptosis of cancer cells is induced by increased ROS generation [10,11]. Because of this, ROS are a promising drug target in cancer therapeutics.

In the present study, we investigated the effects of LCB on Ox-sensitive and -resistant CRC cells (HCT116 and HCT116-OxR cells). We demonstrated, for the first time, that LCB potentiates apoptosis through the ROS-dependent JNK/p38 signaling pathway, suggesting that ROS are a potential therapeutic target for overcoming Ox resistance and that LCB may be a novel therapeutic agent for Ox-sensitive and -resistant CRC treatment.

## 2. Materials and Methods

### 2.1. Cell Lines and Cell Culture

Human colorectal cancer cell line HCT116, mouse epidermal cell line JB6, and human keratinocytes cell line HaCaT were purchased from American Type Culture Collection (ATCC; Manassas, VA, USA). The HCT116, JB6, and HaCaT cells were maintained in RPMI-1640 (GIBCO, Grand Island, NY, USA), MEM (GIBCO), and DMEM media (GIBCO) supplemented with 5% or 10% fetal bovine serum (GIBCO) and 1% penicillin/streptomycin (p/s; Hyclone, Logan, UT, USA). The oxaliplatin-resistant (OxR) colorectal cancer cell line (HCT116-OxR) was obtained from The University of Texas MD Anderson Cancer Center [17]. HCT116-OxR cells were cultured in MEM medium supplemented with 10% FBS, 1% p/s, 1% MEM non-essential amino acids solution, 1% sodium pyruvate, and 1% MEM vitamin solution. All cells were maintained in a 37 °C incubator with a 5% humidified atmosphere of CO_2_/95% air.

### 2.2. Chemical Treatment

Licochalcone B (LCB; purity of 95%) was obtained from Goo Yoon [18]. HCT116 and HCT116-OxR cells were treated with 0, 10, 20, and 30 μM of LCB or 2 μM of Ox for 24 or 48 h. Inhibitors such as SP600125 (JNK inhibitor, 4 μM), SB203580 (p38 inhibitor, 8 μM), NAC (ROS inhibitor, 4 mM), and Z-VAD-FMK (pan-caspase inhibitor, 5 μM) were pretreated for 3 h prior to LCB (30 μM) treatment for 48 h.

### 2.3. Cell Viability Assay

The viabilities of HCT116, HCT116-OxR, JB6, and HaCaT cells were measured by the MTT assay. Briefly, HCT116 (5.5 × 10^3^ cells/100 μL), HCT116-OxR (4 × 10^3^ cells/100 μL), JB6 (8 × 10^3^ cells/100 μL), and HaCaT (8 × 10^3^ cells/100 μL) cells were seeded in 96-well plates. After incubation for 24 h, the cells were exposed to 100 μL of culture medium containing various concentrations of LCB (0, 10, 20, and 30 μM) and Ox (2 μM) for 48 h. In the case of the inhibitor experiment, the inhibitor (4 μM of SP600125, 8 μM of SB203580, 4 mM of NAC, and 5 μM of Z-VAD-FMK) was pretreated for 3 h before LCB (30 μM) treatment. MTT reagent was added to each well. After 40–60 min of reaction at 37 °C, the absorbance density of each well was recorded at 570 nm using a Microplate Spectrophotometer (Thermo Fisher Scientific, Vantaa, Finland).

### 2.4. Soft Agar Assay

For anchorage-independent growth assays, HCT116 and HCT116-OxR cells (8000 cells/well) were mixed with culture medium (including Basal Medium Eagle, 10% FBS, 5 μg/mL gentamicin, 2 mM _L_-glutamine) containing 0.3% agar on 6-well plates with a bottom layer of solidified 0.6% agar in the culture medium. Culture-medium-mixed agar was treated with the indicated concentration of LCB or 2 μM Ox as the positive control. Cultures for each cell line were maintained for 7–10 days at 37 °C in an atmosphere of 5% CO_2_. After 7–10 days, colonies of at least 50 μM in diameter were counted. Images were captured and measured using IMT i-solution software (IMT i-solution Inc, Vancouver, BC, Canada).

### 2.5. Annexin V/7-Aminoactinomycin D Staining

HCT116 (a density of 1.5 × 10^5^ cells/well) and HCT116-OxR (a density of 1.2 × 10^5^ cells/well) cells were seeded in 6-well plates. The cells were treated with LCB (0, 10, 20, and 30 μM) for 48 h or pretreated with 4 mM NAC for 3 h, and then treated with 30 μM LCB for 48 h. At 48 h after treatments, the cells were harvested from the cell culture medium and washed with PBS, followed by the addition of 120 μL of Muse^®^ Annexin V & Dead Cell Reagent (Muse^®^ Annexin V & Dead Cell Kit, Luminex, Austin, TX, USA, MCH100105). The cells were then determined using a Muse^®^ Cell Analyzer system (Merck Millipore, Darmstadt, Germany).

### 2.6. Cell Cycle Analysis

HCT116 and HCT116-OxR cells were seeded into 6-well plates and treated with LCB at various concentrations for 48 h. Following 48 h of incubation, the cells were harvested and then fixed with 70% ethanol at −20 °C for overnight. The ethanol was removed. The cells were washed with cold PBS and added with 150 μL Muse^®^ cell cycle reagent (Muse^®^ Cell Cycle Kit, Luminex, MCH100106) for 30 min in the dark at room temperature (RT). Phases of the cell cycle including sub-G1, G0/G1, S, and G2/M were detected using a Muse^®^ Cell Analyzer system (Merck Millipore).

### 2.7. Analysis of Reactive Oxygen Species (ROS)

The levels of ROS generation were measured using a Muse Oxidative Stress kit (Luminex, MCH100111). HCT116 and HCT116-OxR cells were treated with a Muse^®^ Oxidative Stress Reagent working solution and incubated for 30 min in the 37 °C incubator in the dark. ROS levels were analyzed using a Muse^®^ Cell Analyzer system (Merck Millipore, Darmstadt, Germany) flow cytometer.

### 2.8. Mitochondrial Membrane Potential (MMP) Assay

To evaluate the changes in MMP, CRC cells were cultured in 6-well plates and treated with LCB of 0, 10, 20, and 30 μM concentrations. The cells were harvested and washed with PBS. The cells were incubated with Muse^®^ MitoPotential working solution (Muse Mitopotential Kit, Luminex, MCH100110) at 37 °C for 20 min. Following incubation, samples were added with 5 μL of 7-aminoactinomycin D (7-AAD) and incubated at RT for 10 min. MMP depolarization cells were assessed using the Muse^®^ Cell Analyzer system (Merck Millipore).

### 2.9. Caspase Activity Assay

Multi-caspase activities were measured by the Muse^®^ Multi-caspase kit (Luminex, MCH100109) according to the manufacture’s recommendations. Briefly, HCT116 and HCT116-OxR cells were seeded and treated with DMSO or the indicated concentration of LCB for 48 h. After treatment, the cells were resuspended in caspase buffer. An amount of 50 μL of the cells was transferred to 1.5 mL microcentrifuge tubes and added to 10 μL of Muse^®^ Multi-caspase reagent working solution. Then, the samples were incubated for 30 min in the 37 °C incubator and added to 125 μL of Muse^®^ caspase 7-AAD working solution and incubated in the dark for 5 min at RT. The multi-caspase activity of samples was determined using a Muse^®^ Cell Analyzer system (Merck Millipore).

### 2.10. Western Blot Analysis

The LCB-treated cells were collected and washed with PBS, then added with ice-cold RIPA buffer (iNtRON Biotechnology, Seongnam, Gyeonggido, Republic of Korea) with phenylmethyl sulfonyl fluoride, leupeptin, and aprotinin. The protein concentration was determined using the Bio-Rad Protein Assay kit (Bio-Rad Laboratories, Hercules, CA, USA). Samples with an equal amount of protein (up to 40 μg) were fractionated on 8–15% SDS-polyacrylamide gels, transferred to polyvinylidene difluoride membranes (Millipore, Billerica, MA, USA), and blocked in 3–5% skim milk in PBS containing 1% Tween-20 (PBST) for 2 h at RT. Membranes were washed twice for 5 min and incubated with primary antibody at a 1:1000 dilution for 2 h at RT, followed by overnight incubation at 4 °C. The following primary antibodies were used for Western blots: anti-p-JNK (Cat. #9255), anti-JNK (Cat. #9252), anti-p-p38 (Cat. #9211), anti-p38 (Cat. #9212), anti-Bim (Cat. #2933), and anti-caspase 7 (Cat. #9492) from Cell Signaling (Danvers, MA, USA); anti-β-actin (Cat. sc-47778), anti-p21 (Cat. sc-6246), anti-p27 (Cat. sc-56338), anti-cyclcin B1 (Cat. sc-7393), anti-cdc2 (Cat. sc-8395), anti-GRP78 (Cat. sc-1050), anti-CHOP (Cat. sc-7351), anti-Mcl-1 (Cat. sc-819), anti-Bid (Cat. sc-56025), anti-Bax (Cat. sc-20067), anti-Bcl-xL (Cat. sc-8392), anti-Bcl-2 (Cat. sc-7382), anti-cytochrome c (anti-cyto c Cat. sc-13156), anti-β-tubulin (Cat. sc-166729), anti-COX4 (Cat. sc-69359), anti-Apaf-1 (Cat. sc-33870), anti-caspase 3 (Cat. sc-7148), and anti-PARP (Cat. sc-8007) from Santa Cruz Biotechnology (Santa Cruz, CA, USA). After washing the membranes with PBST, incubations with the secondary antibodies (anti-goat diluted 1:5000, anti-rabbit diluted 1:10,000, and anti-mouse diluted 1:10,000) were conducted for 2 h at RT. Protein bands were visualized with ImageQuant LAS500 (GE Healthcare, Uppsala, Sweden) using Western blotting luminol reagent (Santa Cruz) and quantified by Image J software (National Institutes of Health, Bethesda, MD, USA).

### 2.11. Statistical Analysis

All data values are expressed as means ± standard deviation (SD). Statistical analysis was performed using one-way and two-way analysis of variance (ANOVA) followed by Tukey’s post hoc comparisons. The results were analyzed using GraphPad Software version 5.0 software (San Diego, CA, USA). The *p*-values of less than 0.05, 0.01, and 0.001 were considered statistically significant.

## 3. Results

### 3.1. LCB Inhibits CRC Cell Viability and Colony Formation Ability

**Figure 1:**
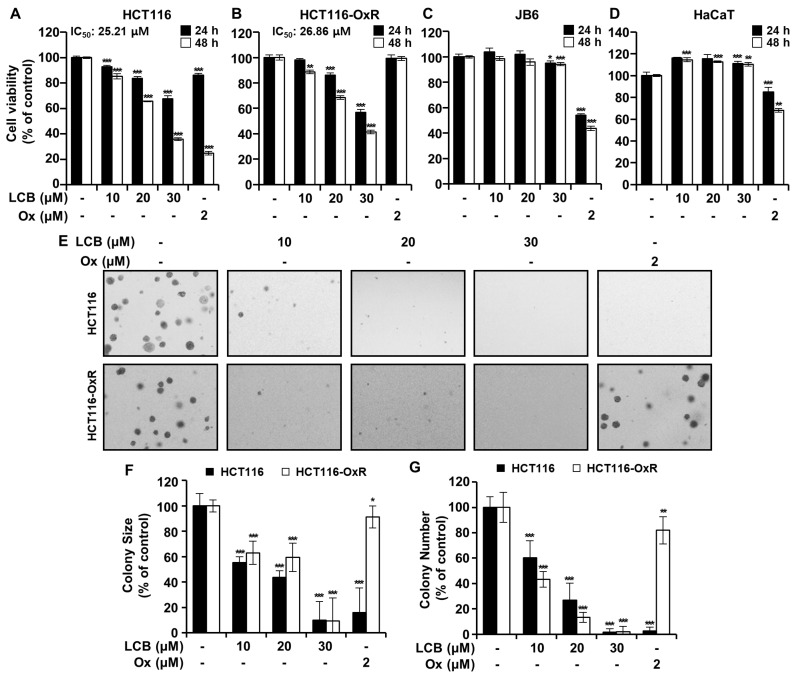
Licochalcone B (LCB) decreases cell viability and restricts the clonogenic properties of oxaliplatin (Ox)-sensitive or -resistant colorectal cancer (CRC) cell lines. HCT116 (**A**), HCT116-Ox-resistant (OxR) (**B**), JB6 (**C**), and HaCaT (**D**) cells were treated or not treated with different concentrations of Ox (2 μM) or LCB (10, 20, and 30 μM) for 24 h or 48 h. The MTT assay was used to reveal the cell viability of CRC cells at 24 h or 48 h after treatment with LCB at various concentrations. (**E**) The anchorage-independent growth ability of CRC cells was assessed using a soft agar assay. The cells were untreated or treated with the indicated concentrations of LCB or Ox and incubated for 7 to 10 days. Bar graphs indicate the colony size (**F**) and numbers (**G**). Colonies > 5 μm were counted and measured. All data are represented as mean ± SD from triplicate experiments (* *p* < 0.05, ** *p* < 0.01, *** *p* < 0.001 versus the untreated group).

The effects of LCB on HCT116 and HCT116-OxR cell proliferation were determined using an MTT assay. As shown in Figure 1A,B, the viability of HCT116 and HCT116-OxR cells was inhibited by LCB treatment in a dose- and time-dependent manner. Cell viability after treatment with LCB (10–30 μM, 48 h) decreased by between 92.61 and 35.70% for HCT116 cells and 96.78 and 41.51% for HCT116-OxR cells (Figure 1A,B). When exposed to LCB for 48 h, the IC_50_ values in HCT116 and HCT116-OxR cells were 25.21 µM and 26.86 µM, respectively. As shown in Figure 1A,B, the viability of HCT116-OxR cells was higher than that of HCT116 cells when they were exposed to an equal concentration of Ox. These results indicate that the HCT116-OxR cell line was resistant to Ox. In addition, LCB did not affect the viability of JB6 or HaCaT cells (Figure 1C,D). A soft agar assay was used to assess the ability to survive in an anchorage-independent manner after treatment with LCB. Figure 1E–G show that LCB treatment significantly reduced anchorage-independent survival and proliferation of HCT116 and HCT116-OxR cells in vitro, and decreased colony size (Figure 1F) and numbers (Figure 1G) in a concentration-dependent manner. When exposed to Ox, the colonies formed by HCT116 cells were smaller and fewer than those formed by HCT116-OxR cells. These findings indicate that LCB inhibited cell viability and anchorage-independent growth in Ox-sensitive and -resistant CRC cells.

### 3.2. LCB Induces Apoptosis by Activating the JNK/ p38 MAPK Signaling Pathway in CRC Cells

**Figure 2:**
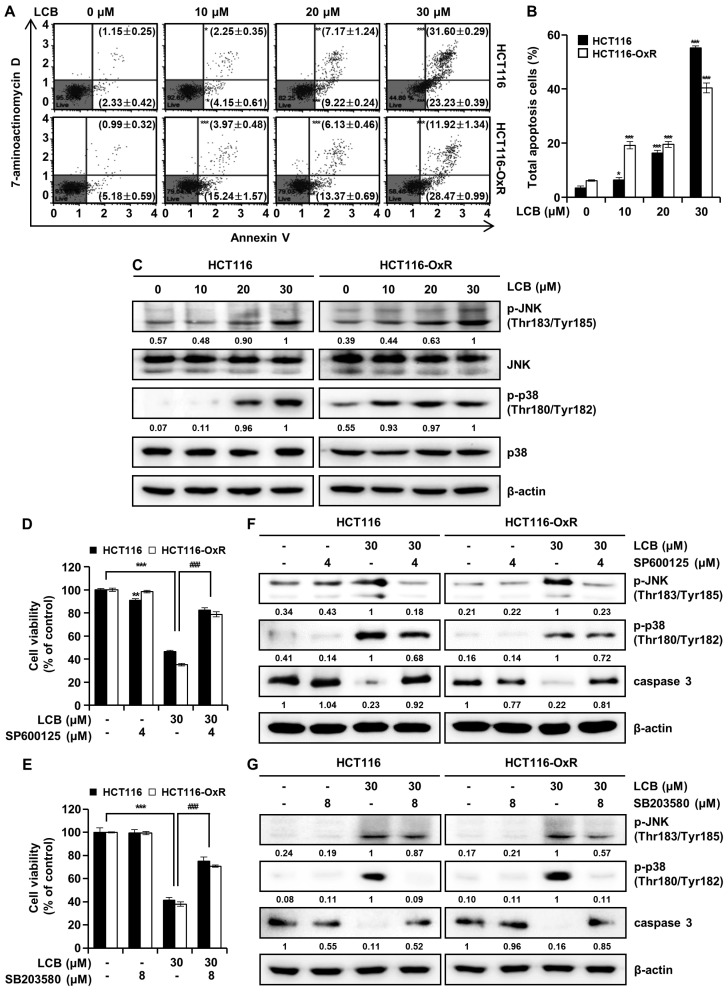
LCB induces apoptosis and causes phosphorylation of JNK/p38 MAPK in human CRC cells. Cells were treated with LCB at 0, 10, 20, and 30 μM for 48 h. (**A**) Annexin V/7-aminoactinomycin D (7-AAD) flow cytometry analysis of CRC cells. Early and late apoptotic cells are represented in the lower right quadrant (Annexin-V-positive and 7-AAD-negative) and upper right quadrant (Annexin-V-positive and 7-AAD-positive), respectively. (**B**) Percentages of early and late apoptotic cells after LCB treatment of CRC cells. (**C**) Western blot showing the expression levels of p-JNK, JNK, p-p38, and p38 in CRC cells. β-actin served as a housekeeping protein. (**D**,**E**) MTT assay was performed to determine the effects of SP600125 (JNK inhibitor) and SB203580 (p38 MAPK inhibitor) on the viability of CRC cells. (**F**,**G**) HCT116 and HCT116-OxR cells were pre-treated with SP600125 (4 μM) or SB203580 (8 μM) for 3 h and then treated with LCB (30 μM) for an additional 48 h. The expression of p-JNK, p-p38, and caspase 3 was examined by Western blot analysis. Results are expressed as the mean ± SD. * *p* < 0.05, ** *p* < 0.01, *** *p* < 0.001 compared with the control group. ### *p* < 0.001 compared with the LCB-alone-treated group.

To evaluate whether the cell-growth-inhibitory effects of LCB are caused by the induction of apoptosis in CRC cells, cells stained with the Muse^®^ Annexin V & Dead Cell Kit were subjected to flow cytometric analysis. The assay results showed that exposure to LCB (48 h) increased the percentage of apoptotic cells in a concentration-dependent manner compared with that in untreated controls. As shown in Figure 2A,B, compared with that in the control groups, the total apoptosis rate increased from 3.48 ± 0.66% to 55.22 ± 0.68% in HCT116 cells and from 6.17 ± 0.27% to 40.39 ± 1.84% in HCT116-OxR cells after treatment with 30 μM LCB for 48 h. To investigate whether JNK and p38 MAPK signaling are involved in apoptosis induced by LCB, their expression was examined by Western blot analysis. Figure 2C shows that LCB clearly induced phosphorylation of JNK and p38 MAPK in a dose-dependent manner. These data confirm that JNK and p38 MAPK are mediators of LCB-induced apoptosis. To determine whether the JNK/p38 MAPK pathway is required for LCB-induced apoptosis in CRC cells, we added inhibitors SP600125 and SB203580 targeting JNK and p38 MAPK, respectively, prior to LCB treatment. As illustrated in Figure 2D, pretreatment with the JNK inhibitor (SP600125) effectively suppressed LCB-induced cell inhibition of cell proliferation in HCT116 (LCB, 46.39 ± 1.17%; SP600125 + LCB, 82.44 ± 2.05%) and HCT116-OxR (LCB, 35.04 ± 1.12%; SP600125 + LCB, 78.80 ± 2.08%) cells. As shown in Figure 2E, pretreatment with the p38 MAPK inhibitor (SB203580) decreased LCB-induced inhibition of cell viability in HCT116 (LCB, 41.48 ± 2.30%; SB203580 + LCB, 75.07 ± 3.48%) and HCT116-OxR (LCB, 37.97 ± 1.97%; SB203580 + LCB, 70.73 ± 0.92%) cells. LCB induced the phosphorylation of JNK, but co-treatment with the JNK inhibitor SP600125 reduced p-JNK (Figure 2F). There was little change in the trend of p-p38 between LCB-treated cells and SP600125 co-treated cells. As shown in Figure 2G, LCB upregulated the phosphorylation of p38, but a decrease in the level of p-p38 was observed in cells co-treated with a p38 MAPK inhibitor (SB203580). There was little change in p-JNK expression between LCB-alone-treated cells and SB203580 co-treated cells. The expression of caspase 3 was decreased in the LCB alone treatment group, and the SP600125 or SB203580 combination treatment group showed similar trends to the control group (Figure 2F,G). These results reveal that LCB-induced apoptosis was dependent on the JNK/p38 MAPK signaling pathway.

### 3.3. LCB Led to CRC Cell Cycle Arrest and ROS Generation

**Figure 3:**
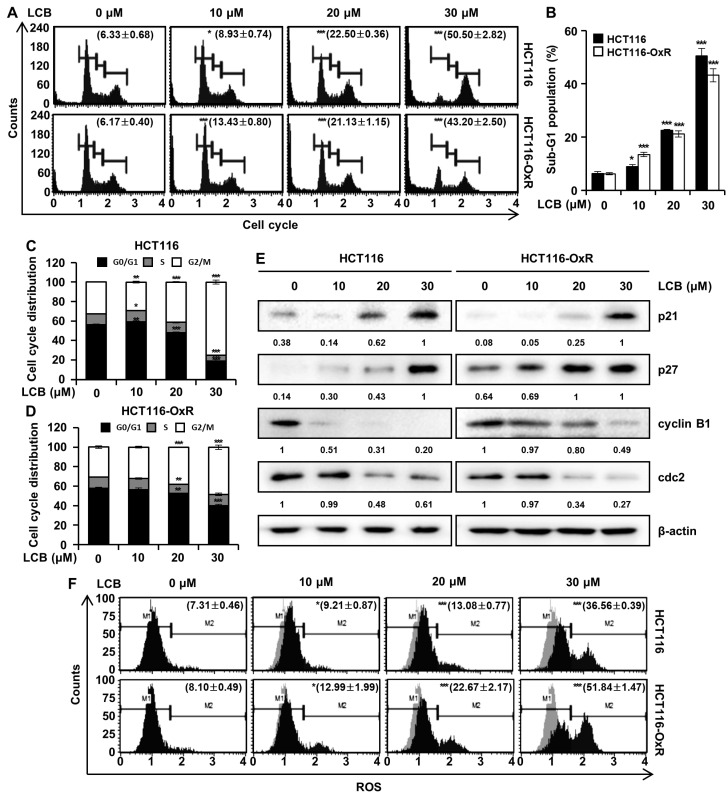
LCB induced G2/M cell cycle arrest in CRC cells. HCT116 and HCT116-OxR cells were treated with LCB for 48 h. (**A**) Cell cycle was detected by flow cytometry. (**B**) Bar-graphical representation of the sub-G1 population. (**C**,**D**) Cell cycle distribution of HCT116 and HCT116-OxR cells by flow cytometry. (**E**) Western blotting with antibodies to determine cell-cycle-related proteins, including cyclin B1, cdc2, p21, and p27. β-actin was used to confirm equal loading of protein samples. (**F**) ROS production was detected by flow cytometry after treatment of CRC cells with LCB for 48 h. * *p* < 0.05, ** *p* < 0.01, *** *p* < 0.001 versus the untreated control.

The effect of LCB on the cell cycle was determined to elucidate the mechanism of action of LCB in CRC cells. As shown in Figure 3A,B, following treatment with 0, 10, 20, and 30 μM LCB, the percentages of the sub-G1 phase in HCT116 cells were 6.13 ± 0.23%, 8.67 ± 0.49%, 14.93 ± 1.21%, and 36.47 ± 2.60%, and those in HCT116-OxR cells were 6.17 ± 0.40%, 13.43 ± 0.80%, 21.13 ± 1.15%, and 43.20 ± 2.50%, respectively. The proportion of G2/M cells increased in a dose-dependent manner in HCT116 and HCT116-OxR cells, indicating that LCB induced G2/M phase arrest (Figure 3C,D). Further, the percentage of HCT116 cells in the G2/M phase increased from 30.50% (control) to 35.57% (LCB 30 μM) (Figure 3C), while that of HCT116-OxR cells increased from 30.73% (control) to 48.50% (LCB 30 μM) (Figure 3D). To further investigate the effect of LCB on the cell cycle distribution, we examined cell-cycle-related protein expression by Western blotting. Figure 3E shows that LCB upregulated the expression levels of p21 and p27 but downregulated cyclin B1 and cdc2. Increased intracellular levels of ROS can lead to the apoptosis of cancer cells [15]. Therefore, we examined the intracellular levels of ROS after LCB treatment using the Muse^®^ Oxidative Stress Kit. ROS levels were increased by 7.31 ± 0.46% (0 μM), 9.21 ± 0.87% (10 μM), 13.08 ± 0.77% (20 μM), and 36.56 ± 0.39% (30 μM) in HCT116 cells and by 8.10 ± 0.49% (0 μM), 12.99 ± 1.99% (10 μM), 22.67 ± 2.17% (20 μM), and 51.84 ± 1.47% (30 μM) in HCT116-OxR cells (Figure 3F). These results demonstrate that LCB induces G2/M phase arrest, which is accompanied by a change in the expression of G2/M-phase-related proteins and upregulates the cellular generation of ROS in CRC cells.

### 3.4. LCB Induces Apoptosis by Decreasing Mitochondrial Membrane Potential (MMP) and Regulating Mitochondrial-Related Proteins

**Figure 4:**
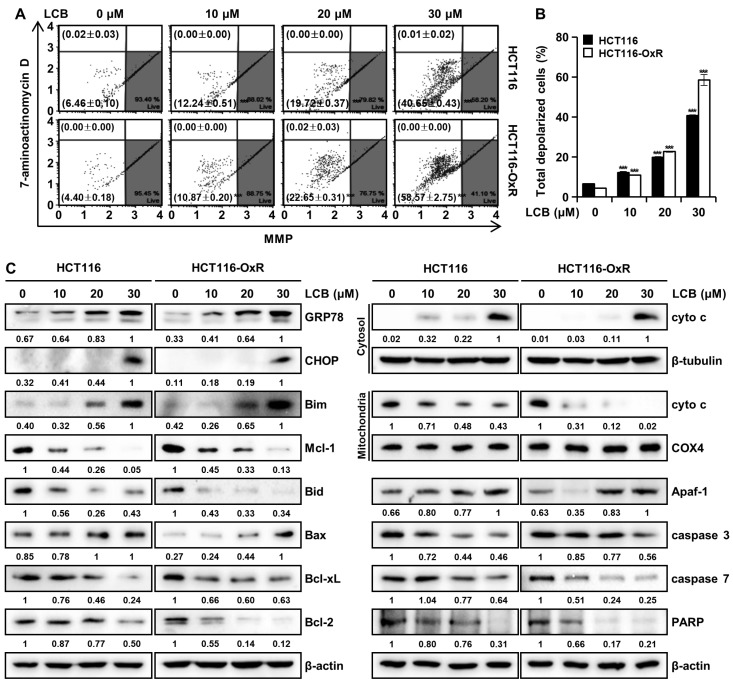
LCB induces apoptosis in CRC cells by disturbing the mitochondrial membrane potential (MMP) and regulating endoplasmic reticulum (ER) stress and apoptosis-related proteins. Cells were treated with 0, 10, 20, and 30 μM LCB for 48 h. (**A**,**B**) Flow cytometry analysis of MMP using the Muse^®^ MitoPotential Kit. *** *p* < 0.001 versus the control group. (**C**) Western blot analysis of ER-stress- and apoptosis-related proteins in CRC cells. Effect of LCB on the expression of GRP78, CHOP, Bim, Mcl-1, Bid, Bax, Bcl-xL, Bcl-2, cytochrome c (cyto c), β-tubulin, COX4, Apaf-1, caspase 3, caspase 7, and PARP proteins. β-tubulin, COX4, and β-actin were used as internal controls. Protein bands were quantified using Image J software.

The expression levels of ER stress- and apoptosis-associated proteins were measured using Western blotting. As shown in Figure 4C, Western blotting showed that compared with those in the DMSO group, the expression levels of ER-stress-associated proteins (GRP78 and CHOP) were markedly increased in the LCB-treated group. Anti-apoptotic proteins such as Mcl-1, Bcl-2, and Bcl-xL inhibit apoptosis by interacting with and isolating pro-apoptotic family members such as Bim and Bax [19]. Truncated Bid translocates into the mitochondria and activates Bax channels, leading to the release of cytochrome c (cyto c) into the cytosol [19,20]. The released cyto c combines with Apaf-1 and caspase 9 to form an apoptosome, which cleaves and activates caspase 3 and caspase 7 [19,20]. Herein, LCB significantly increased the expression of Bim, Bax, cytosolic cyto c, and Apaf-1, but reduced the expression of Mcl-1, Bid, Bcl-2, Bcl-xL, mitochondrial cyto c, PARP, caspase 3, and caspase 7 (Figure 4C). These results demonstrate that LCB treatment induces apoptosis in CRC cells through ER stress and activation of the mitochondrial pathway.

### 3.5. LCB Promotes Multi-Caspase Activity in CRC Cells

**Figure 5:**
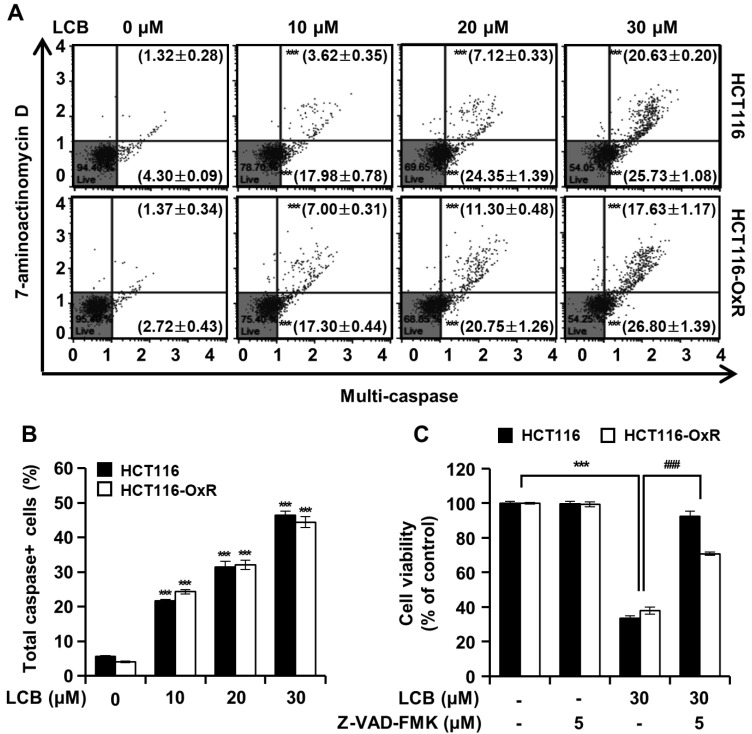
LCB induces multi-caspase activation in CRC cells. CRC cells were treated with indicated concentrations of LCB. (**A**) Activities of multi-caspase were analyzed using the Muse^®^ MultiCaspase Kit (Part Number: MCH100109). Lower-left quadrant: viable cells (caspase-negative and dead-cell-marker-negative). Lower-right quadrant: cells exhibiting caspase activity (caspase-positive and dead-cell-marker-negative). Upper-right quadrant: cells in the late stages of activated caspase or dead (caspase-positive and dead-cell-marker-positive). Upper-left quadrant: dead cells (caspase-negative and dead-cell-marker-positive). (**B**) Quantification of total caspase activation. (**C**) Effects of Z-VAD-FMK, a pan-caspase inhibitor, on LCB-induced apoptosis in CRC cells. Cells were pretreated with 5 μM Z-VAD-FNK for 3 h, and then treated with 30 μM LCB for 48 h. Cell viability was detected using the MTT assay. Data are presented as mean ± SD (*n* = 3). *** *p* < 0.001 compared with the control, ### *p* < 0.001 compared with the LCB-treated group.

LCB treatment (30 μM) increased the percentage of HCT116 and HCT116-OxR (40.72% and 40.35%, respectively) cells with multi-caspase activation (Figure 5A,B) when compared to that in the corresponding DMSO control. To determine whether LCB-induced apoptosis is dependent on caspase activity, HCT116 and HCT116-OxR cells were incubated with 30 μM LCB in the presence or absence of Z-VAD-FMK, a pan-caspase inhibitor. As shown in Figure 5C, compared with the control group, 30 µM LCB decreased HCT116 and HCT116-OxR cell viability by 66.64 and 62.03%, respectively, while the viability of cells treated with Z-VAD-FMK and LCB only reduced by 7.54 and 26.27%, respectively, compared with that in the control group.

### 3.6. ROS Plays a Critical Role in LCB-Induced Apoptosis of CRC Cells

**Figure 6:**
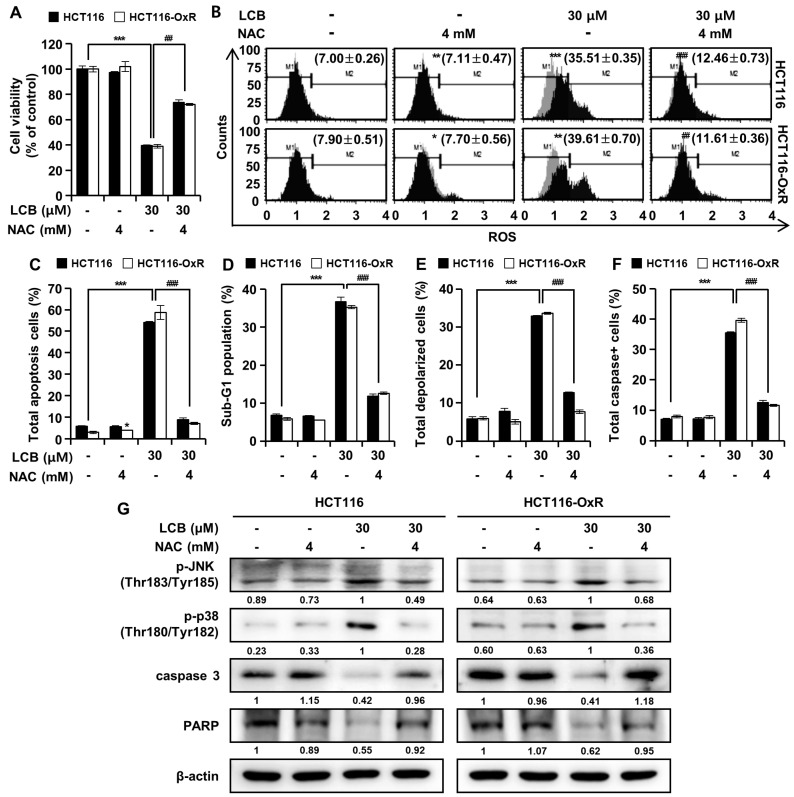
LCB-induced apoptosis is associated with ROS generation in CRC cells. HCT116 and HCT116-OxR cells were pretreated with 4 mM NAC for 3 h and then treated with 30 μM LCB for 48 h. (**A**) Cell viability of HCT116 and HCT116-OxR cells as determined using the MTT assay. Flow cytometry assay analysis of ROS (**B**), apoptosis (**C**), cell cycle (**D**), MMP (**E**), and multi-caspase (**F**) in CRC cells. (**G**) Protein expression of p-JNK, p-p38, caspase 3, and PARP in CRC cells, as determined by Western blot assay. All data are represented as the mean ± SD (*n* = 3), * *p* < 0.05, ** *p* < 0.01, *** *p* < 0.001 compared with the control group (0 μM); ## *p* < 0.01, ### *p* < 0.001 compared with the LCB-treated group (30 μM).

Pretreatment of HCT116 and HCT116-OxR cells with the ROS inhibitor (NAC, 4 mM) for 3 h, followed by exposure to LCB (30 μM) for 48 h, significantly increased cell viability in both cell lines compared to cells treated with LCB only (Figure 6A). Further, the aforementioned procedure was used to determine if LCB-induced ROS may play a role in apoptosis, cell cycle arrest, MMP depolarization, or caspase activation. ROS accumulation was detected in CRC cells after LCB treatment but was abrogated when cells were pretreated with NAC, confirming the effective elimination of LCB-induced ROS production by NAC (Figure 6B). The apoptotic effect was reversed by the addition of NAC, which reduced the apoptotic rate caused by LCB from 54.17 ± 0.33% to 8.82 ± 0.73% in HCT116 cells and 58.80 ± 3.26% to 7.05 ± 0.53% in HCT116-OxR cells (Figure 6C). NAC pretreatment of HCT116 and HCT116-OxR cells inhibited the effects of LCB-induced apoptosis, including the prevention of cell cycle arrest, MMP loss, and multi-caspase activity. ROS have been reported to regulate MAPK signaling [21], and to confirm this, protein expression was examined through a Western blot assay after pretreatment with NAC. The increase in p-JNK and p-p38, as well as the reduction in PARP and caspase 3 levels following LCB treatment, was reversed in the presence of NAC, an ROS scavenger (Figure 6G). As expected, the increase in JNK and p38 MAPK phosphorylation in HCT116 and HCT116-OxR cells was reversed by NAC treatment. These data suggest that LCB induces ROS generation in CRC cells, which, in turn, activates JNK/p38 signaling and induces apoptosis.

## 4. Discussion

Over the past few decades, effective drugs that exhibit anti-tumor effects have been developed. Nonetheless, a major drawback of conventional chemotherapeutic agents is that they affect both cancer cells and healthy tissues in the same manner [2]. In addition, because of the problem of resistance to conventional anticancer drugs, the antitumor effect of these agents is significantly reduced [2,9]. Therefore, to maintain the efficacy of cancer therapy, it is necessary to develop drugs that exert potent cytotoxic effects on tumors and anticancer-drug-resistant tumor tissues, while minimizing toxicity to normal tissues.

CRC is one of the leading causes of cancer-related mortality deaths globally. Until now, the main medical therapy for patients with CRC has been accompanied by several undesirable physiological and immunological side-effects [9]. Ox, a third-generation platinum-based anticancer agent, is used as a first-line treatment for colorectal, gastric, and pancreatic cancers and is undergoing clinical trials for other cancers, including ovarian, breast, and non-small cell lung cancers [22]. Ox is metabolized to oxalate and dichloro(1,2-diaminocyclohexane)platinum (II), which induces excessive neuroexcitation, oxidative stress, and neurodegeneration through several mechanisms, including ion channel dysregulation, mitochondrial dysfunction, neuronal injury, and altered central nervous system function [22]. In addition to these side-effects, resistance to Ox occurs readily and markedly diminishes existing anticancer effects [22]. It has been reported that the acquisition of resistance to Ox was accompanied by cross-resistance to copper influx and efflux transporters such as intracellular p-type ATPases (ATP7A and ATP7B) and the downregulation of human copper transporter 1 (hCTR1) expression [22,23]. Excision repair cross-complementing group 1 (ERCC1), one of the NER mediators, and its catalytic partner, xeroderma pigmentosum group F (XPF, ERCC4), have been demonstrated to be closely related with Ox resistance [24]. Additionally, other proteins of the NER system, such as XPF and xeroderma pigmentosum group G (XPG, ERCC5), have been reported to play a role in Ox resistance [22,24]. Therefore, our study focused on providing new strategies for the treatment or prevention of human Ox-sensitive and Ox-resistant CRC.

As one of the most widely studied herbal medicines worldwide, licorice (*Glycyrrhiza inflata*) root is used to treat various ailments such as arthritis, heart disease, and lung disease [25]. A number of biologically active compounds have been isolated from the plant [25,26]. Herein, we clarified the molecular action mechanism of LCB, a natural compound derived from the roots of *Glycyrrhiza inflata*, through identifying its molecular target. Our study results demonstrated that the cytotoxicity effect of Ox significantly decreased cell viability in HCT116, JB6, and HaCaT cells. In contrast, Ox treatment of HCT116-OxR cells resulted in insignificant cytotoxicity, demonstrating that HCT116-OxR cells were resistant to Ox. A previous report on LCB showed that 0–20 μM LCB treatment had no effect on the normal osteoblast hFOB 1.19 cell line [27]. Furthermore, LCB treatment at concentrations below 50 μM in the murine hepatocyte BRL cell line showed no cytotoxicity and protected hepatocytes from alcohol-induced hepatotoxicity [28]. LCB was not toxic to mouse skin epithelial JB6 cells and human skin keratinocyte HaCaT cells at a high concentration (30 µM) and had an antiproliferative effect on human Ox-sensitive and resistant CRC cells. Therefore, LCB showed no toxicity in normal cells compared to existing anticancer agents and demonstrated anticancer efficacy through significant cytotoxicity in human Ox-sensitive and -resistant CRC cells.

LCB has been reported to inhibit anchorage-independent proliferation and induce apoptosis in oral and non-small cell lung cancer cell lines [10,11]. In this study, LCB inhibited colony formation and induced apoptosis in Ox-sensitive and -resistant CRC cells. This indicates that LCB inhibits anchorage-independent proliferation of Ox-sensitive and -resistant CRC cells. LCA, a licorice extract from a similar family, induced ROS-dependent apoptosis in CRC [29]. Another previously reported licorice extract, echinatin, also induced apoptosis via the ROS-mediated JNK/p38 MAPK signaling pathway in CRC [30]. ROS and free radicals have been reported to directly induce DNA damage via oxidized nucleoside bases, such as 8-oxoguanine formation [31]. Elevated intracellular levels of ROS cause mitochondrial DNA strand breaks and DNA degradation, leading to apoptosis [31]. The induction of apoptosis from the accumulation of ROS has been highlighted as a central mechanism responsible for its positive effects [15]. Excessive intracellular ROS are known to trigger apoptosis through induction of the JNK and p38MAPK signaling cascade [15]. Based on these findings, we investigated whether LCB can cause excessive ROS production and activate the JNK/p38 MAPK pathway in Ox-sensitive and -resistant CRC cells. To this end, LCB increased ROS production (Figure 3F) and phosphorylated JNK and p38 MAPK protein levels (Figure 2C). This supports the hypothesis that LCB induces apoptosis via the JNK/p38 MAPK signaling pathway, which is mediated by excessive ROS production. NAC suppressed the effects of LCB. NAC pretreatment increased the cell viability decreased by LCB (Figure 6A) and decreased the percentage of apoptotic cells (Figure 6B), percentage of ROS-positive cells (Figure 6C), and p-JNK/p-p38 expression increased by LCB (Figure 6G). As shown in Figure 2F and G, compared to the LCB-treated cells, little change in p-p38 expression was observed when co-treated with JNK inhibitor (SP600125), and little change in p-JNK expression was observed when co-treated with p38 inhibitor (SB203580). On the other hand, when treated with ROS inhibitors (NAC), the expression of p-JNK and p-p38 was clearly reduced. Thus, we suggest that LCB-triggered apoptosis is mainly mediated by ROS in human Ox-sensitive and -resistant CRC cells.

Progression through the various stages of the cell cycle is regulated by a series of related proteins, cyclins, cyclin-dependent kinases (CDK), and CDK inhibitors [32,33]. Cyclin B expression is a prerequisite for progression from G2 to M and interacts with CDK1 to induce mitosis [32]. Cip/Kip family members, which are CDK inhibitors containing p21^Cip^, p27^Kip^, and p57^Kip^, share a conserved N-terminal domain that binds to cyclins and CDKs [33]. p21^Cip^ and p27^Kip^ are triggered by antimitotic signals or DNA damage and bind to cyclin-CDK complexes to inhibit their catalytic activity and induce cell cycle arrest [33]. Our results suggest that LCB caused cell cycle arrest at the G2/M stage by decreasing the protein levels of cyclin B1 and cdc2 and increasing the protein levels of p21 and p27 (Figure 3). Elevated ROS levels in cancer cells cause oxidative stress, leading to DNA damage and cell cycle arrest [31]. After pretreatment with the ROS inhibitor, NAC, the sub-G1 phase cell ratio decreased (Figure 6D). This result confirmed that LCB induced sub-G1 cell population and G2/M cell cycle arrest via an ROS-mediated pathway.

Apoptosis is an essential process in multicellular organisms that prevents carcinogenesis by eliminating unwanted or unnecessary cells during development or neutralizing harmful cells due to DNA damage [22]. Apoptosis may be triggered by ROS and/or exposure to xenobiotics such as chemotherapy drugs [15,31]. Most apoptotic pathways lead to caspase activation [16]. Caspase-4 and -12 are involved in ER-mediated apoptosis, and apoptotic caspases consist of initiators (such as caspase-2, -8, -9, and -10) and effectors (such as caspase-3, -6, and -7) [16]. Apoptosis consists of an extrinsic pathway, including death receptors, and an intrinsic pathway involving mitochondria or ER [15,21]. ER chaperone proteins such as GRP78 and CHOP are known to promote apoptotic cell death [34]. In this study, we demonstrated that LCB induces ER stress (Figure 4C). ROS cause oxidation of cardiolipin, a type of mitochondrial membrane phospholipid, and depolarization of the mitochondrial membrane, allowing cytochrome c to be released into the cytoplasm [20]. Cytochrome c forms the apoptosome, which activates effector caspases to cause cellular protein cleavage and DNA damage, leading to apoptosis [31]. Proapoptotic BH3-only proteins, such as Bim, Bax, and t-Bid, inhibit their anti-apoptotic properties by binding to the anti-apoptotic Bcl-2 protein [19]. We showed that LCB triggers the regulation of apoptotic proteins and caspase activation via the mitochondrial pathway (Figure 4 and Figure 5). Furthermore, the caspase inhibitor results confirmed that LCB induces apoptosis via a caspase-dependent pathway (Figure 5C). The percentages of both mitochondrial-depolarized cells and multi-caspase-positive cells, increased by LCB treatment, were decreased by NAC (Figure 6E, F). These data show that LCB induces apoptosis by mediating excessive ROS formation in Ox-sensitive and Ox-resistant CRC cells.

## 5. Conclusions

**Figure 7:**
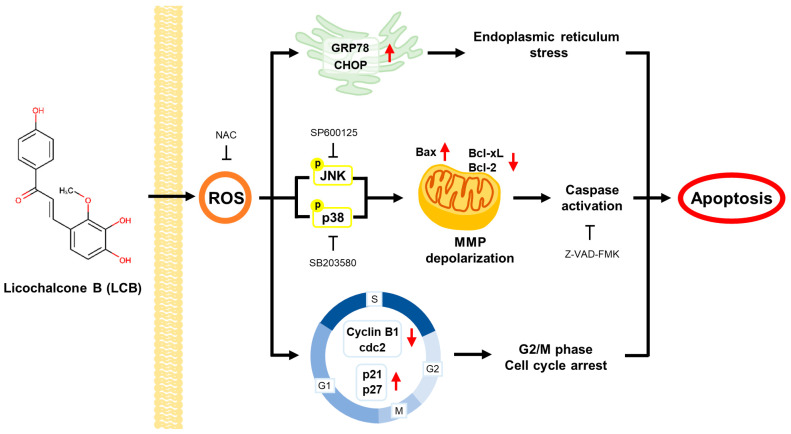
Schematic representation of LCB effects on Ox-sensitive and -resistant CRC cells.

Our study demonstrated that LCB exerts significant in vitro anti-tumor effects in human Ox-sensitive and -resistant CRC through ROS-dependent apoptosis. LCB induces ROS-mediated apoptosis by targeting multiple pathways, including G2/M phase cell cycle arrest, ER stress, mitochondrial dysfunction, caspase activation, and JNK/p38 MAPK signaling (Figure 7). Taken together, our study provides a theoretical basis for the application of LCB in human Ox-sensitive and -resistant CRC. The ability of LCB to modulate ROS-mediated signaling pathways in controlling cancer cells indicates its enormous potential for development as a multi-targeted therapy for Ox-sensitive and -resistant CRC.

## Data Availability

The data presented in this study are available in the article.
